# Targeted degradation of MK2 is insufficient to block inflammatory cytokine production in human cells due to cooperativity with MK3 and MK5

**DOI:** 10.3389/fimmu.2025.1712589

**Published:** 2026-01-14

**Authors:** Bin Yang, Guoqiang Fang, Isaac Marx, Guang Liu, Stephanie Skouras, Kirti Sharma, Dirk M. Walther, Sarah Bollinger Martinez, Cedric Hubeau, Yatao Shi, Chris De Savi, Xin Huang, Annissa Huhn, Rupa Sawant, William R. Proctor, Vaishali S. Dixit, Huijun Dong, Matthew M. Weiss, Nello Mainolfi, Anthony Slavin, Andrew J. Long, Juliet A. Williams, Fergus R. Byrne

**Affiliations:** Kymera Therapeutics, Inc., Watertown, MA, United States

**Keywords:** MK2, MK3, MK5, targeted protein degradation, TLR

## Abstract

Multiple p38 MAP kinase inhibitors have been developed for the treatment of inflammatory diseases such as rheumatoid arthritis, but their effectiveness has been limited due to toxicity and tachyphylaxis, leading to a lack of clinical benefit. Efforts have been made to circumvent this limitation by targeting individual substrates downstream of p38, including MK2 and MK5. This approach has failed to yield clinical benefit despite preclinical evidence of a therapeutic effect. We hypothesized that there is redundancy in the MAPK activating kinase family that would necessitate blocking multiple kinases to sufficiently impact inflammatory processes. We used heterobifunctional protein degraders that either specifically degraded MK2 selectively or degraded MK2/3/5 simultaneously to test the hypothesis, in addition to genetic approaches to enable knockdown. In human PBMCs, elimination of MK2/3/5 with heterobifunctional degraders resulted in full reduction of TLR4 or TLR7/8 induced TNFα, whereas MK2-specific degradation only attenuated TNFα biosynthesis. In contrast, both specific MK2 degradation and broad MK2/3/5 degradation inhibited TGF-β-induced collagen production in human fibroblasts. This observation was consistent with genetic deletions of MK2, MK3 and MK5 (singly and in combination) whereby single deletion of MK2, MK3 or MK5 attenuated lipopolysaccharide (LPS) induced TNFα production and had no effect on R848-induced TNFα production. Double deletion of MK2 and MK3 or MK2 and MK5 or MK2/3/5 triple deletion had a significantly greater effect on TNFα production regardless of stimulus. The combined data suggest cooperativity between MK2 and either MK3 or MK5 for efficient, cell context-dependent modulation of inflammatory responses.

## Graphical Abstract

**Figure:**
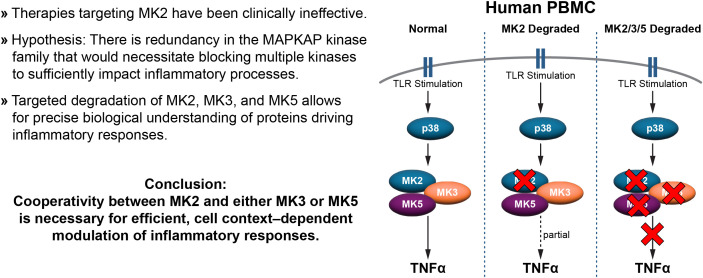

## 1 Introduction

Activation of the p38 mitogen-activated protein kinase (MAPK) pathway has long been known to lead to up-regulated synthesis of pro-inflammatory cytokines in immune cells. Pharmacologic targeting of this signaling axis has been an attractive strategy for the treatment of inflammatory disease such as rheumatoid arthritis. Many p38 kinase inhibitors have been developed that demonstrate inhibition of TNFα, IL-1 and IL-6 production and display anti-inflammatory effects in animal models of inflammation (1). In clinical trials, however, several p38 kinase inhibitors have been discontinued either due to unacceptable safety profiles or the occurrence of tachyphylaxis in which the pharmacodynamic effect on the acute phase reactant, C-reactive protein, was not maintained in the context of continued pharmacologic inhibition of p38 (2). This has been shown to be due to disruption of negative feedback elements where p38 acts as a negative regulator of the upstream kinase TAK1. When p38 phosphorylation is disrupted, negative regulation of TAK1 is released and signaling through the pathway remains intact (3).

Downstream phosphorylation substrates of activated p38 kinase include the mitogen-activated protein kinase-activated protein (MAPKAP) kinases MK2, MK3, and MK5. Targeting of these individual kinases has been a strategy to circumvent some of the pitfalls of p38 inhibition given that they are not involved in the TAK1 feedback loop (4). There is ample evidence that shows MK2 is a compelling target in inflammatory disease. MK2 knockout mice are resistant to collagen induced arthritis in a murine model of rheumatoid arthritis (5) and the pathway has further been linked to roles in neuro-degenerative disease (6), atherosclerosis (7, 8), colon cancer (9, 10), osteoarthritis (11, 12), and lung fibrosis (13, 14). Mechanistically, it has been shown that MK2 can regulate LPS-stimulated TNFα and IL-6 production by impacting stability and translation of mRNAs via AU-rich elements (15, 16). Although this showed that MK2 plays a major role in TNFα biosynthesis, there remained residual production of TNFα, suggesting that an alternative, complementary pathway is operant. Published studies show evidence of cooperativity of members of the MAPKAP kinase family in TNFα production. In mouse knockout studies, MK3 knockout mice showed little reduction in LPS-induced TNFα levels. MK2 knockout mice had significantly reduced TNFα release and the MK2/MK3 double knockout mouse exhibited significantly more inhibition than MK2 knockout mice. This suggests that MK2 is the major MAPKAP kinase regulating TNFα production but does not preclude that MK3 may play an additional role (17). This data, combined with MK2 knockout data in mouse models of inflammation, made MK2 a compelling target for the treatment of inflammatory diseases.

Targeted protein degradation is a novel therapeutic modality that leverages the cellular ubiquitin-proteosome system to selectively degrade disease-causing proteins of interest. Protein degraders can act as functional antagonists by causing degradation of the target to provide a depth of pharmacologic effect deeper than blocking enzymatic activity of the protein alone. This can be true when the protein of interest has scaffolding function independent of kinase activity (18). Protein degraders can be used as effective tools for understanding the functionality of specific proteins given their rapid and robust removal of the protein of interest.

In this study, we leveraged heterobifunctional degraders that either selectively targeted MK2 or were designed to degrade MK2/MK3/MK5. We utilized these tools, in combination with genetic ablation, to probe the relative contribution of the different family members to drive inflammatory cytokine production in human cells. We extend previous mouse studies by showing that members of the MAPK activating protein kinase family, MK2, MK3 and MK5, act cooperatively to effectively transduce TLR4 and TLR7/8 pro-inflammatory signals in human peripheral blood mononuclear cells (PBMCs) and in non-human primates. We also show that the cooperativity is cell context and signal dependent in that MK2, independent of MK3 or MK5, is sufficient to effectively transduce TGF-β production of collagen A1 in fibroblasts.

## 2 Materials and methods

### 2.1 Materials

KTX-0810 and KTX-6222 were synthesized by Kymera Therapeutics. Other reagents are described in respective Method sections (19).

### 2.2 Western blot analysis for MK2 degradation in human PBMCs

Commercialized cryopreserved normal human donor PBMCs (The Primary Cell Solution, #PB100C-W) were obtained from a local vendor. Compounds supplied by Kymera were diluted from a stock solution in a final background concentration of 0.5% dimethyl sulfoxide (DMSO) and incubated as 4.5 x 10^6^ cells/well in a 6-well plate at 37 °C, 5% CO_2_ for 24 hours before extraction into RIPA lysis and extraction buffer (Boston BioProducts, #BP-115D). Proteins were separated by 4-12% SDS-PAGE (Thermo Fisher, #WG1403BOX) and transferred to nitrocellulose membrane (Millipore, #HATF00010).

For Western blot analysis, primary antibodies were 1:1000 dilutions of rabbit monoclonal antibodies to human MK2 (Abcam, #247272), human MK3 (Cell Signaling Technology, #7421) and mouse monoclonal to human MK5 (Santa Cruz Biotechnology, #46667). Mouse monoclonal antibody to actin was from Cell Signaling Technology (#3700), and rabbit monoclonal antibody to actin was from Cell Signaling Technology (#4970). Secondary antibodies were 1:5000 dilutions of IRDye CW goat anti-mouse (LI-COR, #926-68070) or IRDye 800 CW goat anti-rabbit secondary antibody (LI-COR, #926-32211). The blot imaging was performed on an Odyssey CLx imaging system (LI-COR, #9140).

MK2, MK3 or MK5 band intensity were normalized to levels of the actin and represented as percentage of control. Relative half-maximal degradation concentration (DC_50_ values were calculated using the Microsoft Excel Fit software using the formula (Conc Sample –Conc Low Control/Conc High Control –Conc Low Control) * 100%.

### 2.3 Selectivity study in human PBMCs by proteomics

Normal human PBMCs (All Cells, Lot #3076653) from three healthy human donors were cultured in RPMI 1640 media supplemented with 10% fetal bovine serum (FBS). Cells were incubated for 24 hours with a 10-fold concentration of KTX-0810 or KTX-6222 (sufficient to achieve 90% degradation of MK2), or an equivalent volume of DMSO, by adding compounds directly to the culture medium. Global discovery proteomics were performed as described previously (18). Briefly, samples were lysed and digested with trypsin, labeled with 18-plex tandem mass tag (TMT) reagents, pooled, fractionated offline, separated by reversed-phase nano liquid chromatography, and analyzed online with an Orbitrap Eclipse mass spectrometer. Characterization of THP-1 wild-type and MK2/2/5 knockout cells was performed on fractionated, unlabeled peptides using an Orbitrap Lumos mass spectrometer.

### 2.4 LPS or R848-induced TNFα secretion in human PBMCs and human whole blood

Normal human PBMCs from three healthy human donors were obtained from The Primary Cell Solution. Fresh normal human donor whole blood was obtained from a local vendor. Compounds supplied by Kymera were diluted from a stock solution in a final background concentration of 0.5% DMSO and incubated in a 12-well plate at 37 °C, 5% CO_2_ for 20 hours and then LPS (Sigma, #93572-42-0) was added to a final concentration of 0.1 μg/ml for an additional 4 hours and then supernatant was harvested and analyzed for levels of human TNFα by HTRF kit (PerkinElmer CisBio, #62HTNFAPEG), ELISA kit (R&D, #DY210), MSD kit (MSD, #K15345G). For R848 (Invivogen, #tlrl-r848-5), the final concentration in the assay is 1.0 μg/ml.

Relative half-maximal inhibitory concentration (IC_50_) values were calculated using the Microsoft Excel Fit software using the formula (Conc Sample – Conc Low Control/Conc High Control –Conc Low Control) * 100%.

### 2.5 Generation of MK2, MK3, MK5 knockout cells

CRISPR-Cas9 mediated knockout cell clones of MK2, MK3 or MK5—single or combined—in THP-1 cells were generated by Synthego Corporation (Redwood City, CA, USA). To generate these cells, ribonucleoproteins containing the Cas9 protein and synthetic chemically modified sgRNA produced at Synthego were electroporated into the cells using Synthego’s optimized protocol. Editing efficiency was assessed upon recovery, 48 hours post electroporation. Genomic DNA was extracted from a portion of the cells, PCR amplified and sequenced using Sanger sequencing. The resulting chromatograms were processed using Synthego Inference of CRISPR edits (ICE) software (ice.synthego.com).

To create monoclonal cell populations, edited cell pools were seeded at 1 cell/well using a single cell printer into 96 or 384 well plates. All wells were imaged every 3 days to ensure expansion from a single-cell clone. Clonal populations were screened and identified using the PCR-Sanger-ICE genotyping strategy described above.

### 2.6 COL1A1 secretion in BJ fibroblast

On day 1, human BJ fibroblast BJ CRL-2522 cells were seeded into a 96-well plate at 6000 cells/well and, on day 2, cells were starved with EMEM medium with 1% FBS (ATCC, #30–2003 and FBS Gibco, #100911418). On day 3, medium was harvested, and different concentrations of compounds were added for either 1 hour (degrader or degrader control, test agents) or for 20 hours (catalytic inhibitors) in 0.1% DMSO. On day 4, 10 ng/ml of recombinant human TGF-β3 (R&D, #243-B3-010) was added into the cells for 24 hours and on day 5 the supernatant was collected. Levels of type one collagen (COL1A1) were analyzed by ELISA kit (Sangon, #D711146-0096), and relative IC_50_ values were calculated using the Microsoft Excel Fit software using the formula [(Conc Sample – Conc Low Control)/(Conc High Control – Conc Low Control)] * 100%.

### 2.7 LPS-induced TNFα secretion in THP-1 wild type and MK2, MK3 or MK5 knockout cells

On day one, 1 x 10^5^ THP-1 cells/well were plated in 0.1 ml RPMI 1640 medium in 96-well U bottom plates and cells were treated with either DMSO control (final concentration of 0.1%) or MK2 compounds. One hour later the cells were stimulated with 0.1 μg/ml of LPS (Sigma-Aldrich, #L2637) and four hours later the supernatant was harvested and TNFα levels determined by ELISA (R&D systems, kit QK210). Relative IC_50_ values were calculated using GraphPad prism software version 10.2.3. Wild type and knockout THP-1 cells were plated at 1 x 10^5^ cells per well in 100 μl RPMI1640 medium in 96-well U-bottom plates and stimulated with 0.1 μg/ml LPS or 10 μg/ml R848 (InvivoGen, #tlrl-r848). Supernatants were collected after 5 hours, and TNFα production was assessed by ELISA (R&D, #QK210).

### 2.8 LPS-induced TNFα secretion in cynomolgus whole blood

This study in cynomolgus monkeys was conducted in a test facility accredited by the Association for Assessment and Accreditation of Laboratory Animal Care (AAALAC), with oversight by the Institutional Animal Care and Use Committee (IACUC). The study protocol was reviewed and approved by the IACUC, Inotiv, 10424 Middle Mt. Vernon Road, Mt. Vernon, IN 47620, USA.

Cynomolgus monkeys (*Macaca fascicularis*) that were at least 18 months old weighing between 1–4 kg were used in this study. Monkeys (n=3/sex) were dosed via oral gavage with KTX-0810 for 7 days at doses of 2 mg/kg and 30 mg/kg. Whole blood was sampled either pre-dose or at 4 hours post last dose (day 7) to assess MK2 degradation and LPS induced cytokine secretion. Approximately 1 ml of whole blood was collected into each of two TruCulture tubes, one for LPS-stimulated and one for null stimulation, from each animal at each of the following timepoints: Pre-dose (day -3), 4 hours following the day 7 dose and 120 hours following the day 7 dose. For each null tube and each LPS tube, the clarified cell-free supernatant of approximately 500 μl was split on ice into two separate aliquots and stored in a -80°C freezer for subsequent analysis of TNFα levels and compound exposure levels by HPLC/mass spectrometry.

### 2.9 CTG assay in human PBMCs and THP-1 cells

Frozen commercialized human PBMCs were thawed out and recovered at 37 °C in RPMI Medium 1640 (Gibco, # A10491-01) containing 10% FBS (Corning, # 35-081-CV) and 1× penicillin-streptomycin (Solarbio, # P1400) for 2 hrs at 37 °C, 5% CO_2_ incubator. After that, 15,000 PBMC cells were plated into a 384-well plate in 40 μL per well in the growth medium. For THP-1 cells, 2000 cells were plated into a 384-well plate in 40 μL per well in the growth medium in RPMI Medium 1640 containing 10% FBS, 1× penicillin-streptomycin, and 0.05 mM 2-mercaptoethanol (Gibco, #21985023).

For both human PBMC and THP-1 cells, cells were incubated with compounds for 24 hours in the 37 °C, 5% CO_2_ incubator. Then the plates were removed from the incubator and equilibrated to room temperature prior to the addition of an equal volume of CellTiter-Glo^®^ 2.0 reagent. Plates were then shaken at 600 rpm on a plate shaker for 10 minutes at room temperature, while protected from the light. The luminescence signal was read on an Ensignt Instrument (PerkinElmer). The % of inhibition was calculated by the following equation:


$$\%Inhibition=100\%-100\%\times \left(\left(Signal_{sample}-Signal_{LC}\right)/\left(Signal_{HC}-Signal_{LC}\right)\right)$$

Where HC is the signal from DMSO-treated cells; LC is the signal from medium only. Data is representative of n=2 donors (human PBMC) or n=2 experiments (THP-1).

## 3 Results

**Figure 1:**
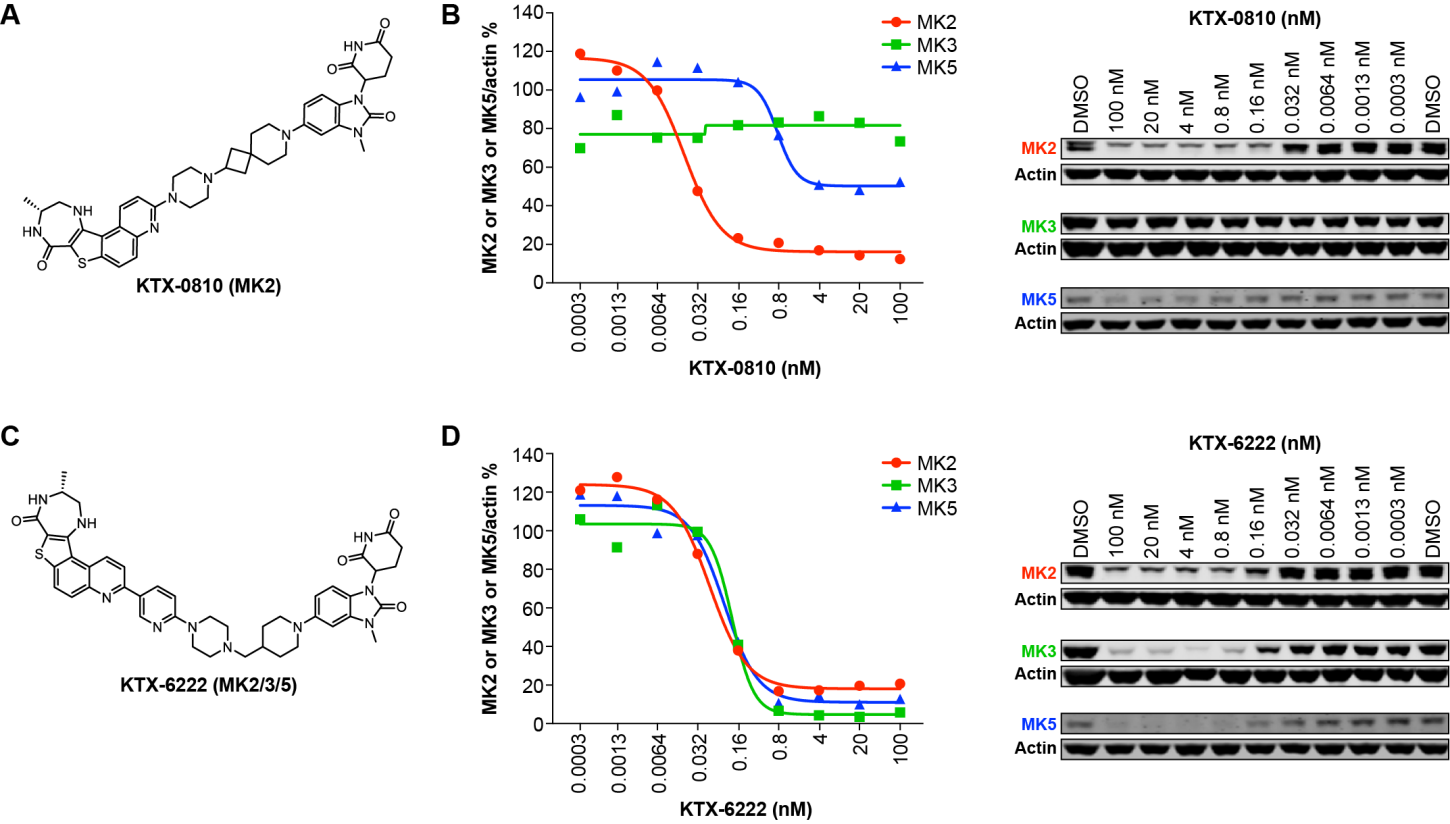
Protein degradation profile of tool compounds in human PBMCs. **(A)** KTX-0810 (MK2). **(B)** KTX-0810 degraded MK2 and partially degraded MK5 while sparing MK3 in unstimulated human PBMCs. **(C)** KTX-6222 (MK2/3/5). **(D)** KTX-6222 degraded MK2, MK3 and MK5 in unstimulated human PBMCs. PBMCs were treated with either DMSO control or degrader compounds for 20 hrs, then stimulated by LPS, and 4 hrs later, the supernatant was harvested, and TNFα levels were determined by ELISA (see Methods). Protein was detected by Western blot. MK2, MK3 or MK5 band intensity was normalized to levels of actin and represented as a percentage of the control. (n=3). Abbreviations: LPS, lipopolysaccharide; MK, mitogen-activated protein kinase-activated protein kinase; PBMC, peripheral blood mononuclear cells.

In this study, we set out to test the hypothesis that there is redundancy in the MAPK pathway that requires multiple family members to be engaged to optimally induce pro-inflammatory cytokines. We used a targeted protein degradation approach to chemically knock down members of the MAPK activated kinase family. Targeted protein degradation is a technology that employs heterobifunctional molecules that bind a protein of interest (i.e. MK2) and bring it in proximity to an E3 ubiquitin ligase that, in turn, leads to protein ubiquitylation and subsequent degradation of the target protein. We designed potent degraders of the MAPK associated kinase family of proteins with differing degradation profiles. These heterobifunctional molecules consisted of a ligand to MK2 that was connected through a linker to a binder that engaged the E3 ligase cereblon. These molecules were identified through design of MK2 degraders that yielded both MK2 selective and MK2/3/5 degraders. To validate that the degraders possessed the properties to test the hypothesis, we evaluated the ability of each compound to degrade MK2/3/5 in unstimulated human PBMCs. KTX-0810 (**Figure 1A**) potently (DC_50_ = 0.02 nM) and effectively degraded MK2 (D_max_ > 90%) and partially degraded MK5 by approximately 50% by Western blot analysis. No degradation of MK3 was observed (**Figure 1B**). The MK2, MK3, and MK5 triple degrader KTX-6222 (**Figure 1C**) potently and robustly degraded MK2, MK3 and MK5 in unstimulated human PBMCs with >90% degradation (**Figure 1D**). The DC_50_ values for MK2, MK3 and MK5 are 0.05 nM, 0.13 nM, and 0.09 nM, respectively. No appreciable degradation of MK2, MK3 or MK5 was observed with a structural analogue of the degraders that lacks the functional capacity for E3 ubiquitin ligase recruitment confirming the requirement of E3 ubiquitin ligase (data not shown). Either KTX-6222 or KTX-0810 did not show any effect on cell viability of human PBMCs up to 1.1 μM (**Supplementary Figure 1**).

To evaluate the ability of these compounds to selectively target the proteins of interest, we performed unbiased discovery proteomics of normal human PBMCs from 3 donors treated with either KTX-0810 or KTX-6222 using tandem mass tag (TMT) quantification. The compounds were tested at a concentration 10-fold over the observed concentration required for 90% protein degradation relative to vehicle (DC_90_ = 0.09 nM). KTX-0810 effectively degraded MK2 with no other proteins in the MAPK pathway being degraded (**Supplementary Figure 2**).

KTX-6222 effectively degraded both MK2 and MK3 while no baseline signal for MK5 was observed in PBMCs due to low abundance of MK5 below the limit of detection. No other proteins in the MAPK pathway were degraded (**Supplementary Figure 2**). KTX-0810 and KTX-6222 were also found to be highly selective across the 468 kinases evaluated in the KINOMEscan screening platform with an S(10) = 0.012 at 100 nM for KTX-0810 and an S(10) = 0.007 at 30 nM for KTX-6222 (**Supplementary Table 1**).

**Figure 2:**
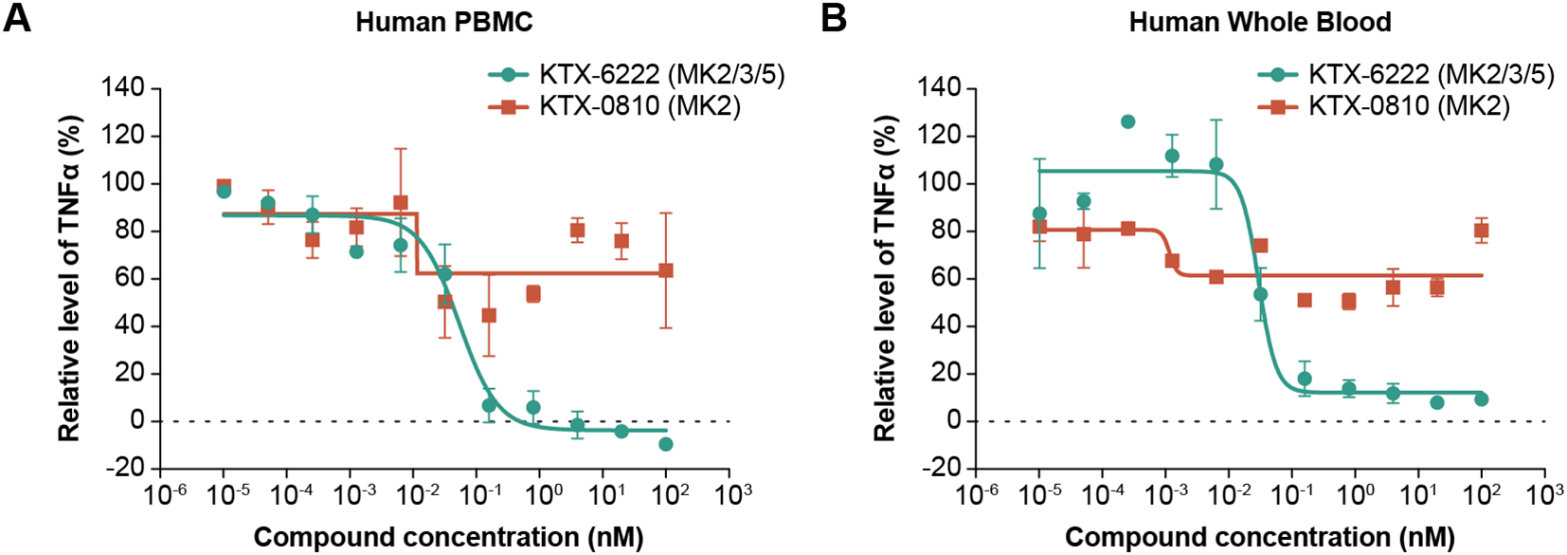
TNFα release from LPS-stimulated PBMCs or whole blood with degrader molecules. **(A)** KTX-0810 (MK2) had no significant effect on LPS-induced TNFα secretion in human PBMCs (orange line), while KTX-6222 (MK2/3/5) potently and effectively inhibited LPS-induced TNFα secretion in human PBMCs (green line). The x-axis represents the concentration of the test agent and the y-axis represents the percent of control for LPS-induced TNFα production relative to no test agent. **(B)** KTX-0810 (MK2) attenuated LPS-induced TNFα secretion in human whole blood (orange line). KTX-6222 (MK2/3/5) potently and effectively inhibited LPS-induced TNFα secretion in human whole blood (green line). Data represents n=3 different donors. Abbreviations: LPS, lipopolysaccharide; MK, mitogen-activated protein kinase-activated protein kinase; PBMC, peripheral blood mononuclear cells.

**Figure 3:**
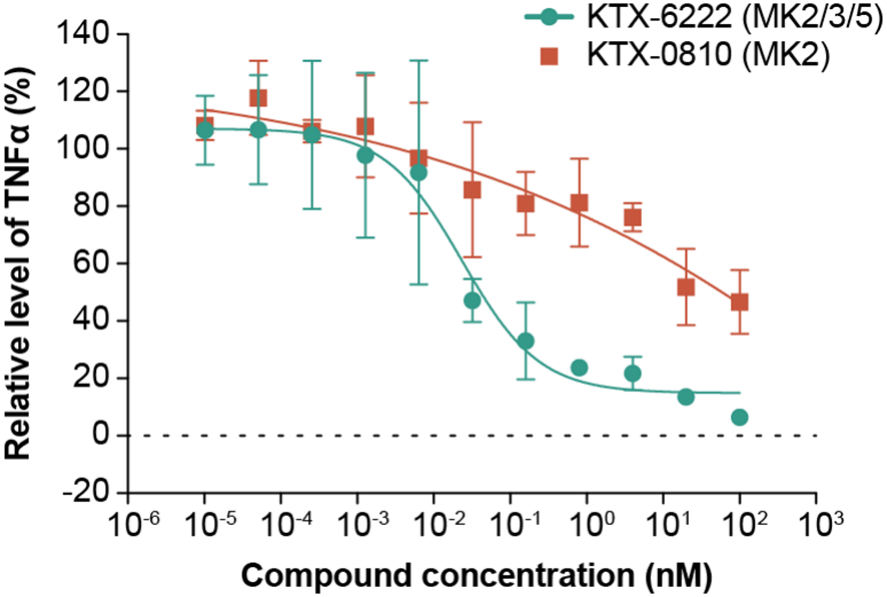
Impact of degrader molecules on TNFα release from R848-stimulated whole blood. The MK2 selective degrader KTX-0810 (MK2) attenuated R848-induced TNFα secretion in human whole blood (orange line). KTX-6222 (MK2/3/5) potently and effectively inhibited R848-induced TNFα secretion (green line). Data represents n=3 different donors. Abbreviation: MK, mitogen-activated protein kinase-activated protein kinase.

Given that both compounds degraded the proteins of interest, we next tested the functional consequence of degrading either MK2 alone or MK2 in combination with MK3 and MK5 on TLR4 dependent TNFα biosynthesis, a pathway known to be dependent on the MAPK activating kinase family. MK2 degradation resulted in significant reduction of LPS induced TNFα secretion in human PBMCs, while MK2/3/5 triple degradation potently and effectively inhibited LPS induced TNFα secretion with IC_50_ of 0.05 nM (**Figure 2A**). A similar effect was observed in whole blood with MK2 degradation resulting in LPS-induced TNFα attenuation, while triple degradation inhibited the response (**Figure 2B**). We also tested the impact of MK2 and MK2/3/5 degradation on R848 driven TLR7/8 signaling, another pathway known to signal through the MAP kinases. Like TLR4-driven responses, MK2 degradation resulted in attenuation of TNFα production in human PBMCs with maximal responses of ~50%. In contrast, triple degradation of MK2/3/5 potently (IC_50_ = 0.02 nM) and effectively inhibited R848 induced TNFα secretion to a greater extent than MK2 degradation alone (**Figure 3**).

**Figure 4:**
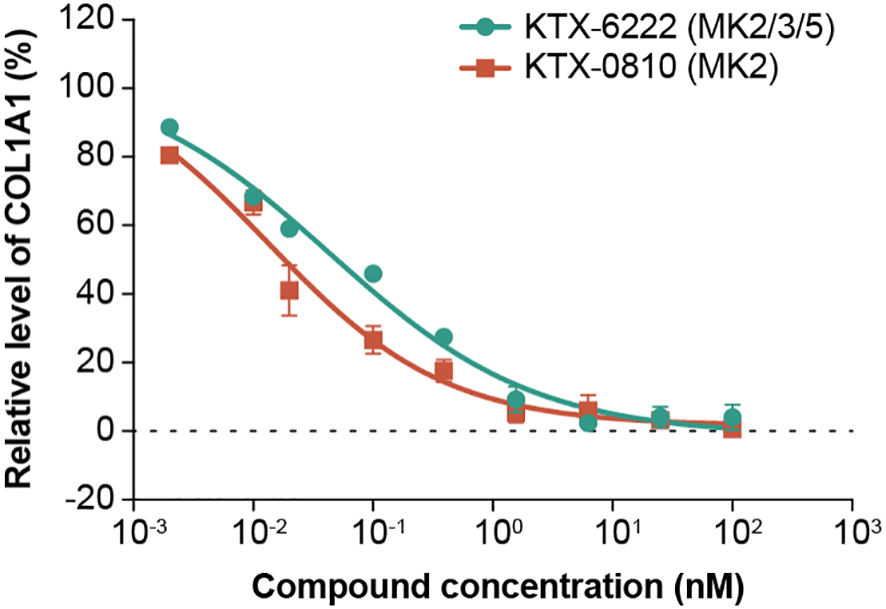
TGF-β-induced COL1A1 release in BJ fibroblasts. KTX-6222 (MK2) and KTX-0810 (MK2/3/5) potently inhibited the TGF- β-induced COL1A1 secretion to a similar extent in the human BJ fibroblast cell line. Data is represented on the Y axis as percent inhibition relative to 10 ng/ml TGF-β3 for 24 hours, with zero test agent for the respective experiment. Abbreviations: COL1A1, type 1 collagen; MK, mitogen-activated protein kinase-activated protein kinase.

It is known that MAPKAP kinase activation is cell and stimulus dependent (20). To address this, we tested the impact of MAPKAP pathway degradation in fibroblasts to determine if there was cooperativity of the different family members in TGF-β driven collagen production. We used the BJ fibroblast cell line stimulated with TGF-β and measured COL1A1 to test the effect of degraders. The production of COL1A1 was inhibited to a similar extent by both MK2 selective and broad MK2/3/5 degradation, indicating that TGF-β response in fibroblasts is exclusively dependent on MK2 and not due to redundant mechanisms or contributions from other MAPK activating protein kinases (**Figure 4**).

**Figure 5:**
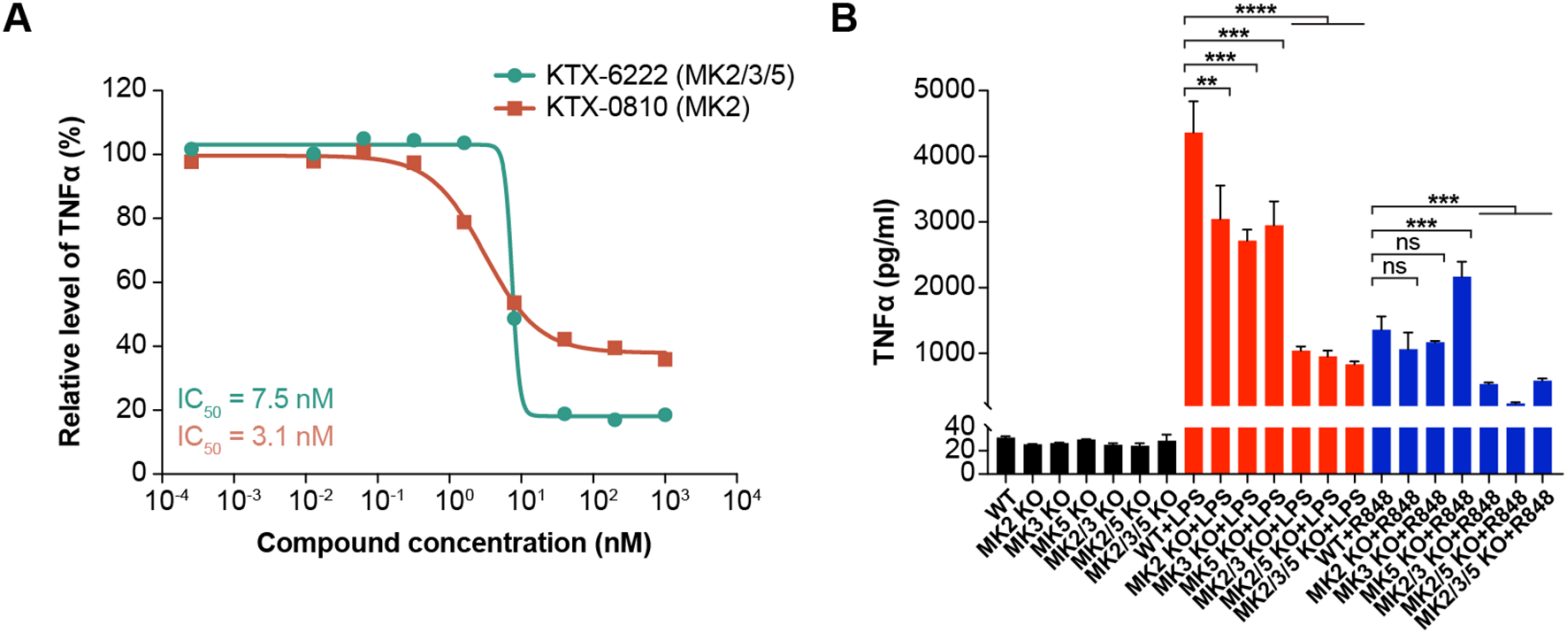
Impact of protein degradation and CRISPR gene editing on LPS-induced TNFα in THP-1 cells. **(A)** KTX-0810 (MK2) partially inhibited LPS-induced TNFα (orange line) while wild-type and knockout cells did not secrete significant amounts of TNFα without stimulation of LPS or R848. KTX-6222 (MK2/3/5) (green line) potently and effectively inhibited LPS-induced TNFα secretion. **(B)** Single deletion of MK2, MK3 or MK5 significantly attenuated LPS-induced TNFα (red bars). Neither MK2 nor MK3 knockout reduced R848 (blue bars) induced TNFα secretion. MK5 knockout significantly augmented R848-driven TNFα biosynthesis. Deletion of MK2/3 or MK2/5 or MK2/3/5 in THP-1 cells inhibited LPS or R848-induced TNFα secretion to a greater extent than single deletion alone. Data is representative of n=3 separate experiments. Statistical analysis between different experimental groups was performed by one-way analysis of variance (ANOVA). Abbreviations: KO, knockout; LPS, lipopolysaccharide; MK, mitogen-activated protein kinase-activated protein kinase; WT, wild type. (*p<0.05; **p<0.01; ***p<0.001; ****p<0.0001; ns, not significant).

Proteomics analysis (**Supplementary Figure 2**) demonstrated that while KTX-0810 and KTX-6222 induced selective degradation within the MAPK pathway (**Figure 1**), they also degraded a limited number of additional proteins. While the other degraded proteins have no known function in the MAPK pathway, we confirmed the specificity of MK2 or MK2/3/5 degradation findings by deleting specific genes using CRISPR-Cas9 knockout in the human monocytic cell line THP-1. In addition to addressing the specificity, CRISPR knockout allowed for modulation of individual kinases to gain a more refined understanding of the contribution of each protein. We first confirmed that the functional effect of MK2 or MK2/3/5 degradation in THP-1 cells to confirm that the cell system was an accurate representation of the degradation profile observed in PBMCs. As shown in **Figure 5A**, pan degrader KTX-6222 markedly inhibited LPS-induced TNFα production by greater than 80% while selective degrader KTX-0810 reduced TNFα production to a lesser extent (62%) with a notably shallower inhibition curve at 10 and 100 nM, respectively. Degradation of the MK2, MK3 or MK5 was confirmed in THP-1 cells and was consistent with PBMC data (data not shown). Next, we used CRISPR-Cas9 gene editing in THP-1 cells to knock out either individual kinases (MK2, MK3, or MK5) or a combination of different kinases in the pathway (MK2/3, MK2/5 or MK2/3/5) to define the requirement and interdependence of different family members in either TLR4 or TLR7/8 dependent processes. Upon LPS stimulation, all single gene knockout cells (MK2, MK3 or MK5) resulted in a significant but attenuated reduction of TNFα compared to wild type THP-1 cells. The reduction of TNFα release in the MK2, MK3, or MK5 knockouts was similar (~30%). The knockout of the combination of kinases (MK2/3, MK2/5 or MK2/3/5) resulted in reductions in TNFα that were significantly greater than the single knockouts alone but were similar to each other (**Figure 5B**, red bars). In response to TLR7/8 stimulation with R848, single knockdown of MK2 or MK3 did not significantly reduce TNFα release. In contrast, knockdown of MK5 significantly increased R848 induced TNFα. Double knockout MK2/3 and MK2/5 and triple knockout MK2/3/5 cells resulted in a significant reduction of R848 induced TNFα that was similar across all combinations, indicating redundancy across members of the family (**Figure 5B**, blue bars). The genes were confirmed to be effectively knocked down by gene sequencing (data not shown), western blot analysis (**Supplementary Figure 3**), as well as proteomic analysis (**Supplementary Figure 4**). To ensure compounds have no effect on THP-1 cell viability, we performed CTG assay in THP-1 cells, which were treated by KTX-6222 or KTX-0810 for 24 hr; no cell toxicity was observed up to 1 μM (**Supplementary Figure 5**).

**Figure 6:**
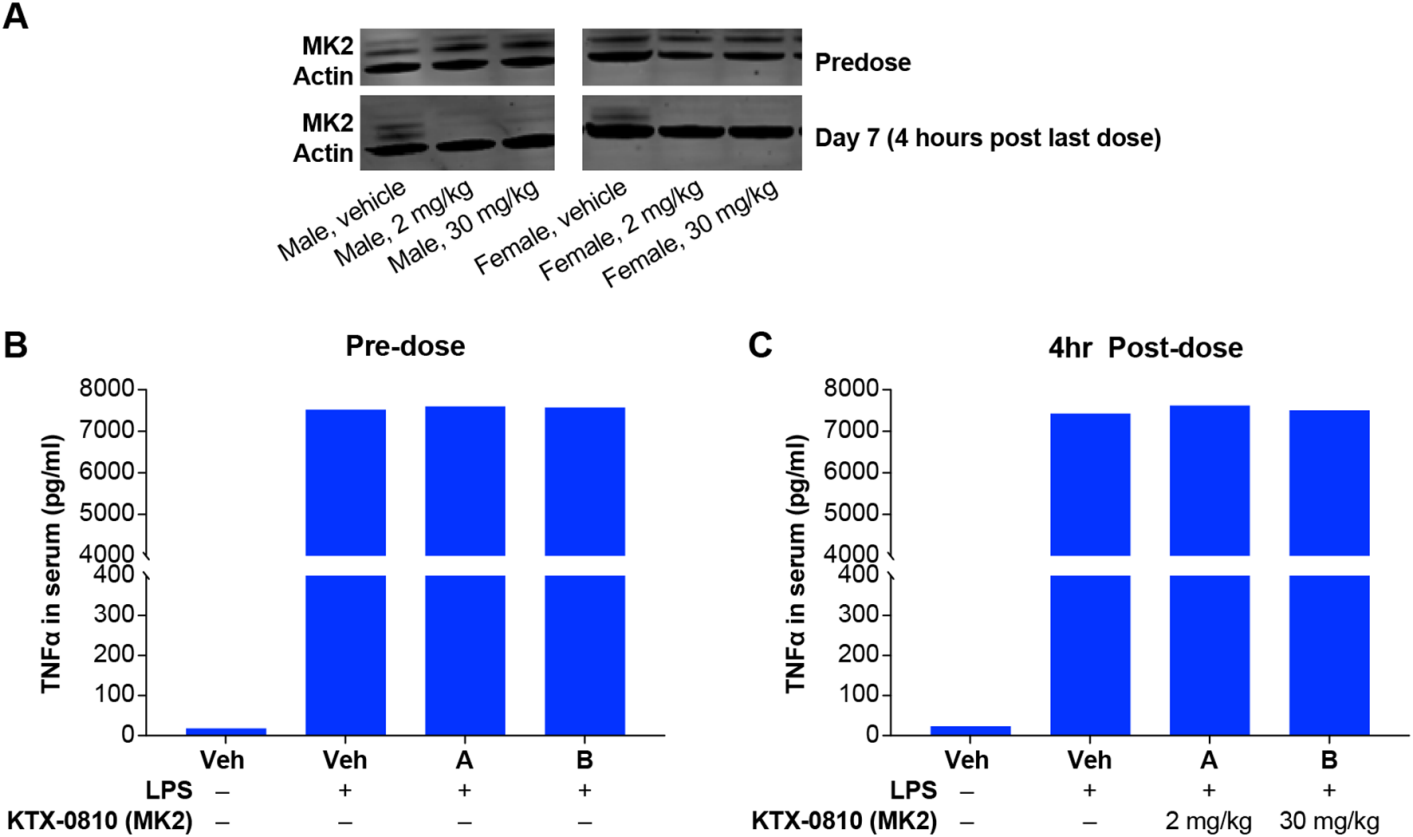
Impact of MK2 degradation in dosed cynomolgus monkeys. **(A)** Western blot analysis of MK2 protein expression from pre-dosed cynomolgus monkeys (top panel) showing intact MK2. MK2 expression is eliminated in samples taken from animals dosed with KTX-0810 (MK2) for seven days, 4 hours post last dose. **(B)** Whole blood from animals prior to dosing shows induction of TNFα in serum after LPS stimulation ex vivo. **(C)** Cynomolgus monkeys were treated with KTX-0810 (MK2) for seven days, and whole blood was collected 4 hours after the last dose and stimulated with LPS. KTX-0810 (MK2) treated animals did not show any reduction in LPS-induced TNFα compared with vehicle controls. Abbreviations: LPS, lipopolysaccharide; MK, mitogen-activated protein kinase-activated protein kinase; Veh, vehicle.

We extended these observations *in vivo* to determine if MK2 degradation in a complex physiological system would recapitulate the findings observed in isolated cellular systems. Non-human primates were dosed with KTX-0810 for 7 days at doses of 2 mg/kg and 30 mg/kg. Whole blood was sampled either pre-dose or at 4 hours post last dose (day 7) to assess MK2 degradation and LPS induced cytokine secretion. After 7 days of dosing, MK2 was effectively degraded compared with pre-dose samples in both male and female animals (**Figure 6A**). *Ex vivo* stimulation of whole blood with LPS from animals prior to dosing resulted in a robust induction of TNFα. Whole blood from animals dosed with KTX-0810 did not show a reduction of TNFα in response to LPS even in the context of robust degradation of MK2. This observation was consistent across dose groups and between sexes (**Figures 6A–C**).

## 4 Discussion

In this study, we tested the hypothesis that redundancy in the MAPK-associated kinase pathway was required for effective transduction of inflammatory stimuli. We designed and evaluated a pair of heterobifunctional degraders that either selectively degraded MK2 or were pan degraders of MK2/MK3/MK5. These degraders are composed of ligands for MK2 tethered through a linker to a ligand for the E3 ubiquitin ligase cereblon. This facilitates ternary complex formation and subsequent ubiquitination and degradation of these proteins. These tools allowed for testing the hypothesis in multiple cellular systems in a rapid and effective way. We established the validity of this approach by demonstrating >90% degradation of MK2 with KTX-0810 in both human PBMCs and whole blood. We also demonstrated robust degradation of MK2, MK3 and MK5 with KTX-6222 (**Figure 1D**). We further illustrated that the selective MK2 degrader KTX-0810 had no effect on TLR4 or TLR7/8 driven TNFα release in human PBMCs or human whole blood, despite degradation of MK2. This data suggests that TLR4 or TLR7/8 driven signals in PBMCs are not completely dependent on MK2 alone. In contrast, the concurrent degradation of MK2/3/5 completely inhibited LPS or R848 induced TNFα production, suggesting a required interplay between the different kinases. This data was also confirmed in the human monocytic cell line THP-1 where MK2 degradation had no effect on TNFα release while MK2/3/5 degradation blocked the response.

The above-mentioned degradation data could imply that MK3 and/or MK5 act as negative regulators of MK2. In this scenario, deleting MK3 or MK5 would remove counter-regulatory mechanisms and allow for efficient and full transduction via MK2. This would be consistent with previous work that described an opposing interplay between MK2 and MK3. Activation of MK2 was shown to regulate LPS-induced IFNβ expression and downstream STAT3 activation. The data suggested that MK3 mediated negative regulatory effects on NF-ĸB and interferon regulatory factor 3 dependent LPS signaling and that activated MK2 restrained the negative regulatory effect (21). Alternatively, the observed degradation data could suggest that either MK3 or MK5, or both, are required for full transduction of TLR4 or TLR7/8 signals in cooperation with MK2. In the current study, the knockout data in THP-1 cells (**Figure 5B**) support this interpretation. When any of the individual MAPK activating protein kinases are knocked out in THP-1 cells, TLR4-driven TNFα release is attenuated similar to the degradation effect. If there were a negative regulatory role of either MK3 or MK5, TNFα release would be augmented rather than attenuated. Combined knockout indicates a cooperativity between MK2 and MK3 or MK5 in the release of TNFα given that the effect is more pronounced than any of the single gene knockouts alone. The combination knockouts of MK2/3, MK2/5 and MK2/3/5 would also suggest that the MK3 and MK5 play redundant roles given that the reduction of TNFα is not increased when the three proteins are deleted compared with either individual combination.

A similar interpretation could be made for TLR7/8 driven responses with some distinct differences. In contrast to TLR4 driven responses, single knockout of any of the genes do not have any impact on TNFα release by themselves except for the MK5 knockout cells which augment the release of TNFα. This would indicate a potential negative regulatory role of MK5 in TLR7/8 dependent TNFα release. Comparison of the combined knockout to the single knockout cells suggests an essential cooperativity of MK2 and MK3 or MK5 in TLR7/8 signaling. Interestingly, TLR4 driven responses in whole blood from cynomolgus monkeys dosed with MK2 degrader showed no effect on TNFα production (**Figure 6**). This may indicate species dependent differences in the pathway utilization as well. This suggests that there is potentially a mouse-human disconnect and that a selective MK2 degrader would not be sufficient in reducing TNFα in humans and reducing TNFα-mediated disease; these findings provide mechanistic insight into the failure of MK2 inhibitors in clinical trials and support multi-target degradation of MAPKAP kinases as a promising strategy to achieve durable anti-inflammatory effects in human disease.

We also observed, in a separate cellular system, that these observations are cell and signal context dependent. The protein degradation studies in BJ fibroblasts showed that TGF-β dependent COL1A1 production is dependent on MK2 given that the magnitude of COL1A1 reduction was similar between MK2 degradation and MK2/3/5 degradation (**Figure 4**). Previous studies have shown a dependence of pro-fibrotic pathways including TGF-β dependent myofibroblast differentiation and actin expression on MK2 (22). This is also consistent with animal model experiments in which bleomycin induced pulmonary fibrosis can be inhibited with a peptide that blocks the function of MK2 (23).

There is precedence for these observations as the complex interplay between MK2, MK3 and MK5 is still emerging. On a molecular level, others have suggested that functional differences between MK2 and MK3 are the consequence of the proline-rich SH3-targeting region in MK2 (24). It has also been described that the phosphorylation of the RNA binding protein tristetraprolin is mediated by MK2 and MK3, which then enables TNFα biosynthesis via stabilization of TNF mRNA (25). However, it was shown that tristetraprolin independent roles of MK2/3 activity also contribute to neutrophil driven responses. It is possible that mechanisms beyond the canonical MAP kinase/NF-ĸB pathway could play a role as it has been shown in dendritic cells that ribosomal S6 kinase is activated by ERK and MK2 and MK3 as well (26). The data presented here also provide some context on the role of MK5 in the transduction of both TLR4 and TLR7/8 driven responses. Shiryaev and Moens (27) previously discussed the evidence both for and against the role of MK5 in MAPK signaling. The interdependence of MK2 and MK3 were also observed in a mouse model of acute proliferative glomerulonephritis with MK2 and/or MK3 knockout mice. The data demonstrated that MK2 and MK3 have redundant and non-overlapping functions. MK2 -/- animals showed minimal impact on mortality while MK3 -/- animals displayed worse survival. Combined MK2 and MK3 knockout animals fared worse than either knockout alone. Differences in survival were suggested to be caused by distinct roles of MK2 and MK3 in the stress response (28). Here, we provide evidence of MK5 cooperativity in TLR4 driven TNFα release and a potential negative regulatory role in TLR7/8 driven responses. More detailed experiments will be needed to further uncover the molecular mechanism.

The interplay of multiple MAPKAP kinase family members may be put in context of clinical data to understand the translatability of our findings to the development of effective therapeutics. Multiple MK2 inhibitors have been developed, including CC-99677 and ATI-450. CC-99677 is a covalent binder of MK2 with biochemical selectivity against p38 isoforms and 364 kinases at their individual K_m_ and cellular selectivity for MK2 (29) while ATI-450 is a selective, reversible MK2 inhibitor that is >700 fold selective over p38-PRAK and p38-ATF (4). Although these compounds differ in their molecular mechanism of inhibition, the functional inhibition of MK2 is similar. These have been tested in both healthy volunteers and in patients. When CC-99677 was dosed in healthy volunteers, whole blood from dosed individuals was stimulated with LPS and TNFα was measured. TNFα release remained reduced over the dosing period, suggesting that targeting MK2 addresses the tachyphylaxis associated with p38 inhibition. However, the magnitude of inhibition of TNFα saturated at approximately 70% at the highest dose tested. The compound also reduced LPS induced TNF release by about 50% in isolated human PBMCs (30). Similar data was generated with ATI-450. Inhibition of TNFα achieved a greater decrease but inhibition of other cytokines in the ex vivo study was variable (31). In a phase 2a study in rheumatoid arthritis patients, modulation of inflammatory biomarkers as well as some evidence of effect on disease measures were observed (32). However, ATI-450 was tested in a larger phase 2 study in moderate to severe rheumatoid arthritis patients where no significant impacts on disease compared with placebo were observed. In addition, a selective MK5 inhibitor (GLPG0259) was developed for the treatment of rheumatoid arthritis. In a phase 2 study in methotrexate refractory patients, GLPG0259 failed to achieve a significant effect on disease measures compared to placebo (33). The combined data, along with the *in vitro* data shown here, may suggest that inhibition of either MK3 or MK5 is needed in addition to MK2 to achieve full suppression of the pathway. Alternatively, the pathway may not play a predominant role in the disease in the patient populations tested. Further work will be needed to confirm if this is the case. The failures of multiple inhibitors puts into question the translatability of preclinical models of inflammation for this pathway. The magnitude of inhibitory effect in the mouse may be misleading for the development of p38 pathway inhibitors.

This complex molecular interplay between p38 MAP kinase, MK2, MK3 and MK5 suggests that selective targeting of a single MAPKAP kinase family may be insufficient to fully blunt inflammatory responses. Hence, a broader modulator that antagonizes or degrades MK2, MK3 and/or MK5 may well be required for the effective suppression of inflammation.

## Data Availability

The raw data supporting the conclusions of this article will be made available by the authors, without undue reservation.
