# Supplementary material for: Targeted degradation of MK2 is insufficient to block inflammatory cytokine production in human cells due to cooperativity with MK3 and MK5
Source: Front Immunol. 2026 Jan 14;16:1712589. doi: 10.3389/fimmu.2025.1712589 (PMC12847452; doi:10.3389/fimmu.2025.1712589)
Supplement: Supplementary file 1 [file DataSheet1.pdf]

Supplementary Material

1 Supplementary Figures and Tables

1.1 Supplementary Table

Compound Name	Selectivity Score Type	Number of Hits Number of Non-Mutant Kinases	Screening Concentration (nM)	Selectivity Score
**KT-0810**	S(35)	5	100	0.012
	S(10)	1	100	0.012
	S(1)	0	100	0
**KT-6222**	S(35)	3	30	0.007
	S(10)	3	30	0.007
	S(1)	1	30	0.002

Supplementary Table 1. KINOMEscan; a high-throughput site-directed competition affinity binding assay against a panel of 468 purified kinases, reveals high selectivity of the MK ligands.

The Selectivity Score or Sscore is a quantitative measure of compound selectivity. It is calculated by dividing the number of kinases that compounds bind to by the total number of distinct kinases tested, excluding mutant variants.

S = Number of hits / Number of assays

This value can be calculated using %Ctrl as a potency threshold (below) and provides a quantitative method of describing compound selectivity to facilitate comparison of different compounds.

- S(35) = (number of non-mutant kinases with %Ctrl <35)/(number of non-mutant kinases tested)
- S(10) = (number of non-mutant kinases with %Ctrl <10)/(number of non-mutant kinases tested)
- S(1) = (number of non-mutant kinases with %Ctrl <1)/(number of non-mutant kinases tested)

1.2 Supplementary Figures

1.2.1 Supplementary Figure 1

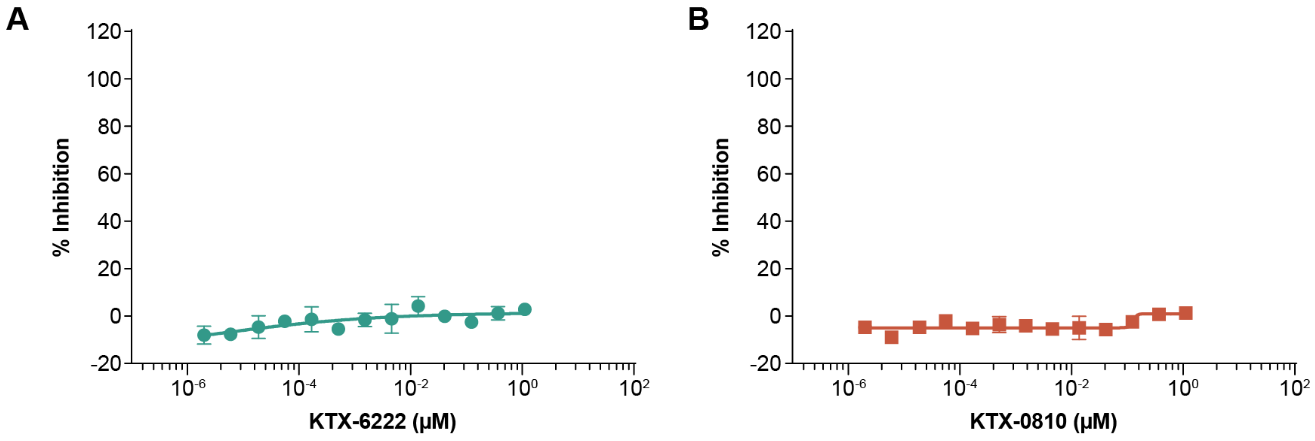

Supplemental Figure 1. KTX-6222 (MK2/3/5) (A) and KTX-0810 (B) have no effect on cell viability in unstimulated human PBMCs. Frozen human PBMCs were thawed and recovered at 37 °C in RPMI Medium 1640 containing 10% FBS and 1× penicillin-streptomycin for 2 hrs prior to incubation with KTX-6222 and KTX-0810 for 24 hrs at 37 °C. Cell viability was determined by CellTiter-Glo 2.0 assay (see Methods). Data is representative of n=2 donors.

1.2.2 Supplementary Figure 2

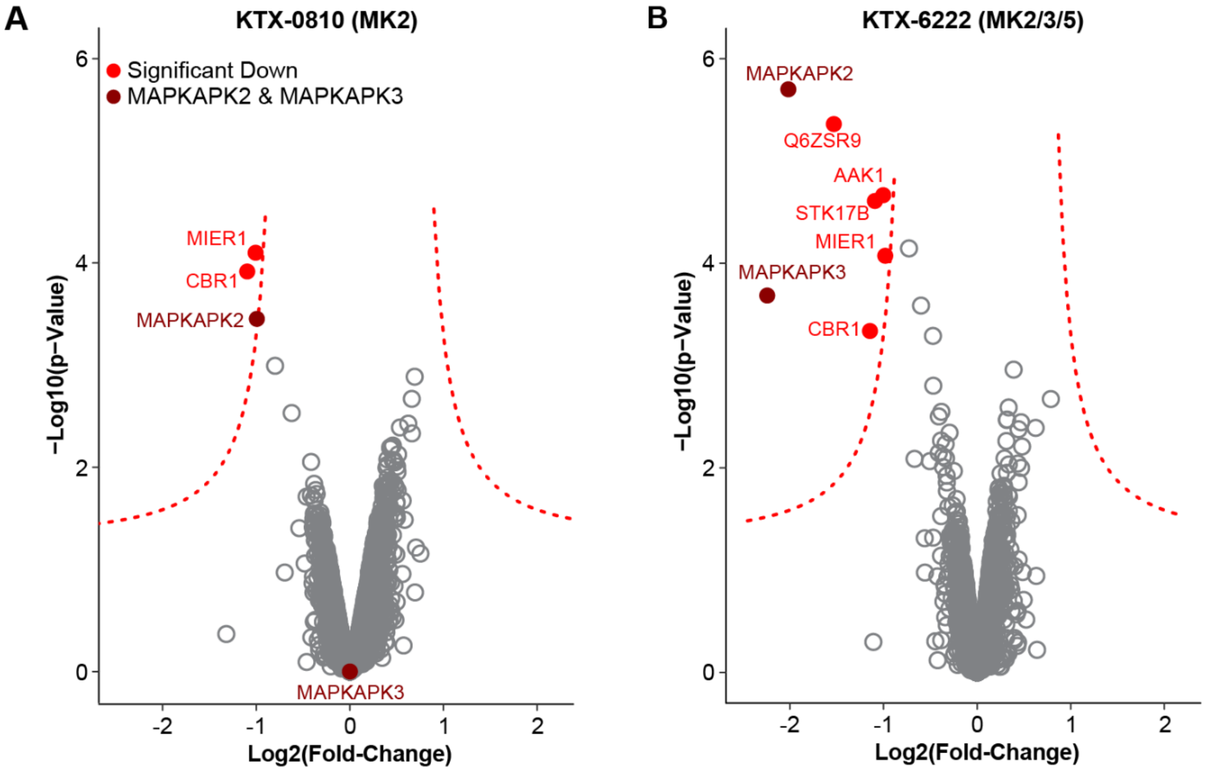

Supplementary Figure 2. Selectivity of tool compounds in human PBMC by proteomic analysis. (A) KTX-0810 (MK2) effectively degraded MK2 in a proteome-wide analysis of >5,400 proteins identified by mass spectrometry in normal, unstimulated human PBMCs. Other proteins degraded similarly to MK2 in PBMCs were mesoderm induction early response protein 1 (MIER1) and carbonyl reductase (CBR1). These proteins have not been shown to play a role in MAPK signaling and are not expected to influence the results. (B) KTX-6222(MK2/3/5) similarly effectively degraded MK2 and MK3 in normal, unstimulated human PBMC. MK5 was not identified due to low expression level and sensitivity of proteomic analysis. The other observed proteins that were degraded with KTX-2622 were MIER1, CBR1, serine/threonine kinase 17b (STKB), adaptor-associated protein kinase 1 (AAK1) and an uncharacterized 37.9 kD protein on human chromosome 2p13.3 (Q6ZSR9).

1.2.3 Supplementary Figure 3

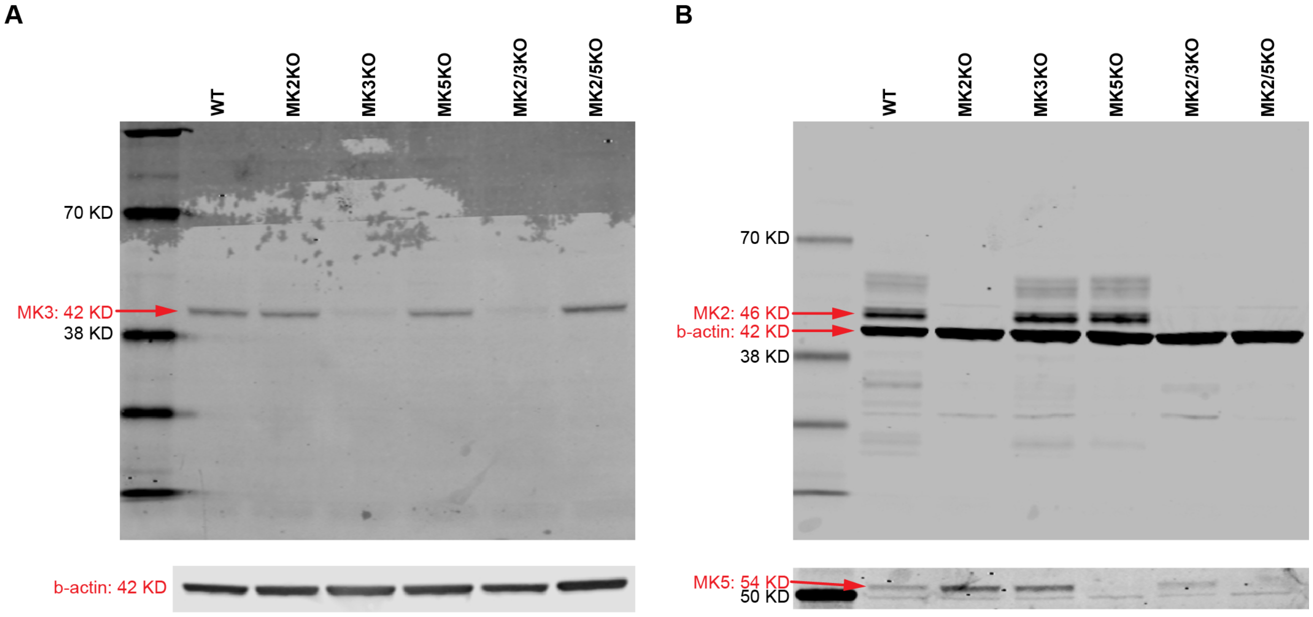

Supplementary Figure 3. Western blotting to confirm the knockout of MK2, MK3, and MK5 in THP-1 cells. THP-1 cells were harvested and washed with PBS and lysed with RIPA buffer with phosphatase and protease inhibitors cocktail. (A) MK3 protein was detected in the wild type (WT), MK2 knockout, MK5 knockout, and the MK2/MK5 double knockout THP-1 cells, but not in the MK3 knockout and the MK2/MK3 double knockout in THP-1 cells. (B) Top: MK2 protein was detected in WT, MK3 knockout and MK5 knockout THP-1 cells, but not in MK2 knockout, MK2, MK3 double knockout, and MK2/MK5 double knockout THP-1 cells. Bottom: MK5 protein was detected in WT, MK2 knockout, MK3 knockout, and MK2/MK3 double knockout cells, but not in MK5 knockout and MK2/MK5 double knockout THP-1 cells.

1.2.4 Supplementary Figure 4

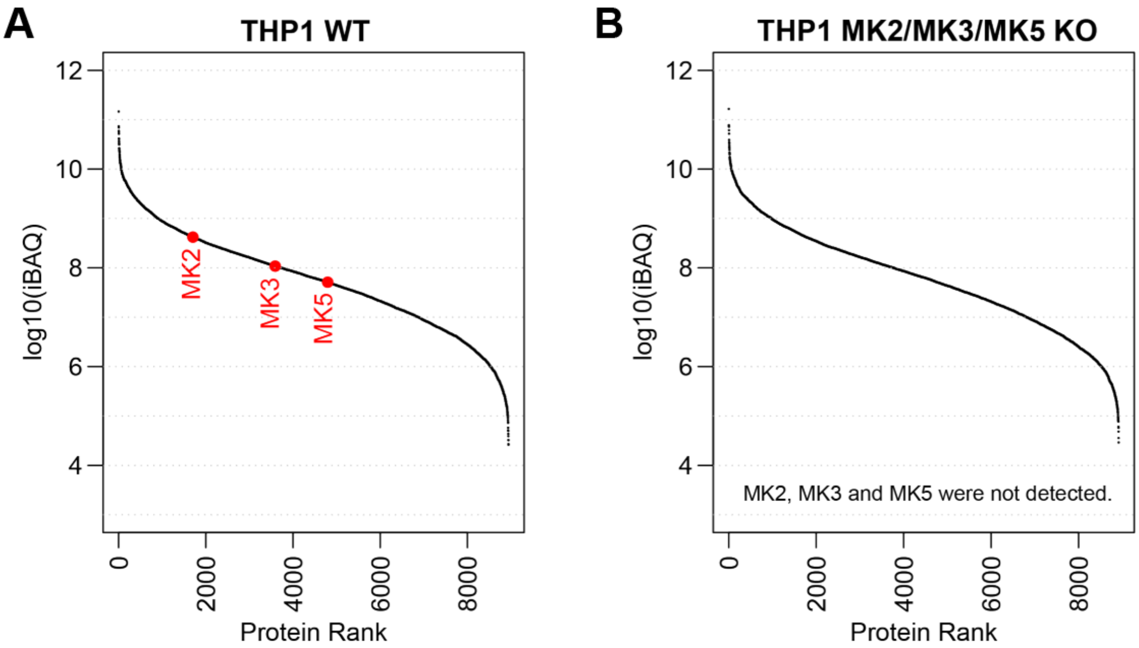

Supplemental Figure 4. (A) Abundance of MK2, MK3, and MK5 in WT THP-1 cells relative to >8,900 proteins quantified using the intensity-based absolute quantification (iBAO) method (1). (B) MK2, MK3, and MK5 were undetectable in the triple knockout cell line despite comparable proteome coverage.

1.2.5 Supplementary Figure 5

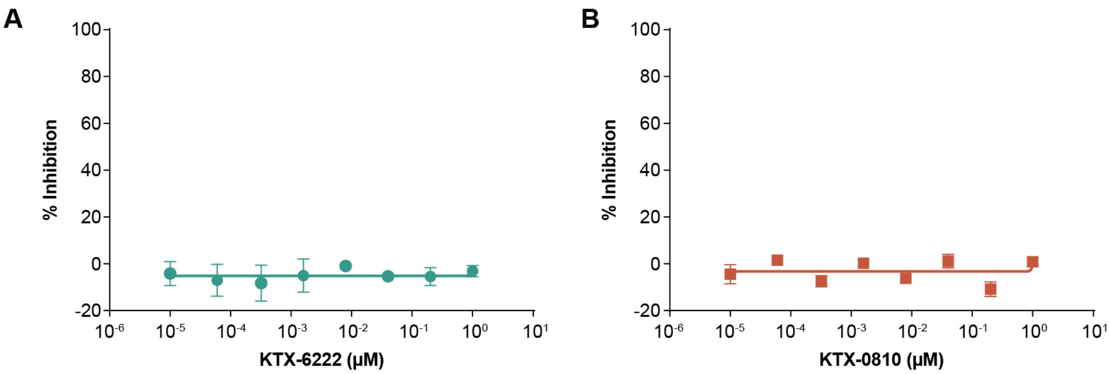

Supplemental Figure 5: KTX-6222 (MK2/3/5) (A) and KTX-0810 (B) have no effect on cell viability in THP-1 cells. THP-1 cells grown in RPMI Medium 1640 containing 10% FBS,1× penicillin-streptomycin and 0.05 mM 2-mercaptoethanol were treated with KTX-6222 and KTX-0810 for 24 hrs at 37°C. Cell viability was determined by CellTiter-Glo 2.0 assay (see Methods). Data is representative of n=2 experiments.

2 Supplementary Reference

1.Schwanhäusser B, Busse D, Li N, Dittmar G, Schuchhardt J, Wolf J, Chen W, Selbach M. Global quantification of mammalian gene expression control. *Nature*. 2011;473(7347):337-342.
